# CD133 Is Associated with Increased Melanoma Cell Survival after Multikinase Inhibition

**DOI:** 10.1155/2019/6486173

**Published:** 2019-07-16

**Authors:** Cynthia M. Simbulan-Rosenthal, Anirudh Gaur, Hengbo Zhou, Maryam AbdusSamad, Qing Qin, Ryan Dougherty, Leala Aljehane, Li-Wei Kuo, Sahar Vakili, Kumari Karna, Havens Clark, Edward McCarron, Dean S. Rosenthal

**Affiliations:** ^1^Department of Biochemistry and Molecular Biology, Georgetown University School of Medicine, Washington, DC 20007, USA; ^2^Division of Surgical Oncology, Harry and Jeanette Weinberg Cancer Institute, MedStar Franklin Square Medical Center, Rosedale, MD, USA

## Abstract

FDA-approved kinase inhibitors are now used for melanoma, including combinations of the MEK inhibitor trametinib, and BRAF inhibitor dabrafenib for BRAFV600 mutations. NRAS-mutated cell lines are also sensitive to MEK inhibition* in vitro*, and NRAS-mutated tumors have also shown partial response to MEK inhibitors. However, melanoma still has high recurrence rates due to subpopulations, sometimes described as “melanoma initiating cells,” resistant to treatment. Since CD133 is a putative cancer stem cell marker for different cancers, associated with decreased survival, we examined resistance of patient-derived CD133(+) and CD133(-) melanoma cells to MAPK inhibitors. Human melanoma cells were exposed to increasing concentrations of trametinib and/or dabrafenib, either before or after separation into CD133(+) and CD133(-) subpopulations. In parental CD133-mixed lines, the percentages of CD133(+) cells increased significantly (p<0.05) after high-dose drug treatment. Presorted CD133(+) cells also exhibited significantly greater (p<0.05) IC50s for single and combination MAPKI treatment. siRNA knockdown revealed a causal relationship between CD133 and drug resistance. Microarray and qRT-PCR analyses revealed that ten of 18 ABC transporter genes were significantly (P<0.05) upregulated in the CD133(+) subpopulation, while inhibition of ABC activity increased sensitivity, suggesting a mechanism for increased drug resistance of CD133(+) cells.

## 1. Introduction

Melanoma is the most dangerous type of skin cancer, accounting for over 96,000 cases and 7,230 deaths in the United States alone. Despite the use of kinase inhibitors, melanoma has high recurrence rates, even after extended latency. Melanomagenesis is associated with driver mutations in the MAPK pathway, including activating changes in codons L597, V600, or K601 of BRAF (*v-raf *murine sarcoma viral oncogene homolog B1; 50% of melanomas) or Q61 of NRAS (neuroblastoma* RAS* viral oncogene homolog; 20%); amplification or activating mutations of C-KIT (2-8%), or LOF mutations in the tumor suppressor NFI (nuclear factor I; 10-20%). These mutations occur in conjunction with changes in other signaling pathways including (1) RAS/PI3K/Akt, (2) p16^Ink4a^/CDK4/Rb, (3) Wnt, and/or (4) p53 [1, 2]. Treatment for* BRAF*-mutant melanoma was the first to improve by targeted therapy using BRAF and MEK inhibitors. Some NRAS-mutated cell lines are also sensitive to MEK inhibition* in vitro *[3], and NRAS-mutated tumors have also shown a partial response to MEK inhibitors [4]. More recently, treatment for* NRAS*-mutant metastatic tumors improved for individuals expressing PDL1, who receive immune-based therapies as first-line treatments, and then chemotherapy with carboplatin, dacarbazine, or temozolomide.

Each of these genetically diverse melanomas can be enriched for highly tumorigenic subpopulations based on assays including (1) melanosphere formation [5], (2) retention of membrane dyes [6], or exclusion of Hoechst dye [7], and (3) expression of receptors, cell adhesion molecules, or other markers including ABCB5 [8], CD20 B lymphocyte antigen [9], CD44 [10], CD133 [11], CD144/Vascular endothelial (VE)-cadherin [12], CD166/activated leukocyte cell adhesion molecule (ALCAM) [13], CD271/low affinity nerve growth factor receptor (LNGFR) [14], aldehyde dehydrogenase 1 (ALDH1) [15], Nestin [16], Tie1 [17, 18], JARID1B H3K4 demethylase [6], or a combination of different markers [8, 11, 19–24]. It was reported that melanoma cells expressing three of these markers: CD133, ABCB5, and/or CD144, form stem cell niches for nonendothelial channels facilitating tumor blood supply in a process termed “vasculogenic mimicry” [23]. Our recent study of 4 markers in various stages of human cutaneous melanoma has shown elevated expression of two of these markers: CD133 and ABCB5, in lymph node and distant organ metastasis [21]. Importantly, a query of the TCGA database using the UCSC Xena Functional Genomics Explorer (https://xenabrowser.net) found a significant negative correlation between CD133 expression and days to death (Kaplan-Meier Estimator; Supplementary Figure s5). The association of CD133 and ABC transporters has been observed by other investigators, who found that CD133(-) overexpressing glioma cells were drug resistant, in part due to the induction of ABCB1 expression and activity [25].

CD133 (Prominin-1) is a pentaspan membrane glycoprotein expressed in a variety of tissues. It appears to play a role in stem cell DNA repair, survival, proliferation, and attachment to cadherins. While its ligand is unknown, it responds to Wnt and TGF*β* superfamily signaling (*e.g., *bone morphogenetic protein 4; BMP4) and has been implicated in normal retinal development in humans and mice. In humans, missense mutations, nonsense mutations, and frameshift-inducing deletions have been associated with disorders related to retinal degeneration, including retinitis pigmentosa (RP4) and macular degeneration (STGD4, MCDR2, and CORD1 [26–28]). A number of studies have suggested that CD133 is associated with stem cells in normal renewing tissues, including hematopoietic [29], epidermal [30], and intestinal stem cells [31]. CD133 has also been shown to be a potential marker of stem cells of different cancers including those of the brain [32], ovary [33], liver [34], prostate [35], pancreas [36], and colon [37, 38].

CD133 is also believed to be a marker of melanoma stem cells, although the finding that this can be model specific (mouse strain,* etc. *[39]) has resulted in adopting the term “melanoma initiating cells” (MIC). In contrast to the aforementioned studies, and in likely disparity with other hematologic and solid malignancies, various reports have demonstrated that approximately one-fourth of single unselected melanoma cells can initiate tumors in severely immunocompromised NOD-SCID-Il2R*γ*^−/−^ (NSG) mice. This high frequency might be considered to be inconsistent with a stable cancer stem cell model of melanomagenesis [6, 39–41] and support the idea that melanomas possess microenvironment-regulated phenotypic plasticity that reverts even highly aggressive malignant phenotypes [42–45]. Subsequent findings showed that such plasticity, requiring widespread alterations in gene expression, is due to epigenetic reprogramming involving alterations in microRNA expression and global chromatin remodeling [46–50].

Whether due to the differential survival of a stable stem cell subpopulation or a microenvironment-induced epigenetic switch, subsequent recurrences and metastases linked with MIC [21] are unfortunately fatal. Presumably, many available cancer chemotherapeutics have invariably failed to eliminate these small MIC populations due to the expression of drug resistance or other survival genes. The surviving epigenetically semistable MIC populations expand and are targets for additional genetically stable driver mutations. In support of this idea, we showed that CD133 positivity was correlated with recurrent patient disease, poor clinical outcomes, and decreased overall survival. Further, we showed that CD133-positive cells isolated from patient tumors formed tumors in nude mouse xenografts, whereas CD133-negative cells did not [21].

In the current study, we analyzed the potential roles of CD133 in chemoresistance. CD133(+) cells showed increased chemoresistance compared to CD133(-) cells sorted by either FACS or MACS. Mixing CD133(+) and CD133(-) cells confirmed these findings and suggests a cell autonomous (nonjuxtacrine or nonparacrine) mechanism. siRNA knockdown of CD133 increased sensitivity to trametinib (T) and dabrafenib (D). Microarray analysis suggests that upregulation of ABC efflux pumps may mediate the CD133 response, since siRNA-mediated CD133 knockdown reduces ABCG2 expression, and inhibition of ABCG2 resensitizes CD133(+) cells. Together, these results suggest that the CD133-ABCG2 pathway is an attractive target for intervention in melanoma.

## 2. Materials and Methods

### 2.1. Cells

Cells were isolated from fresh lymph node human melanoma metastases from patients with poor clinical outcomes: STU (BRAF^V600K^), BAK (NRAS^Q61K^), and BUL (NRAS^Q61K^). Suspensions were prepared by repeated mincing in Iscove's medium containing 10% FBS and 1% penicillin-streptomycin and analyzed for melanoma antigens MART1 and S100 by immunofluorescence microscopy. Cells were maintained in IMDM with 10% FBS and 1% penicillin-streptomycin in a 37°C 5% CO_2_ humidified incubator and passaged 1:4 at 80% confluency. To maintain similar population doublings, large numbers of cells were routinely frozen, and the presence of BRAF^V600^ or NRAS^Q61^ mutations was verified by Sanger sequencing (Supplemental Figures s1 A-C).

### 2.2. Plasmids, Transduction, and Selection

Cells were transduced with pLHCX-DsRed or GFP retroviral vectors using the *ϕ*NX retroviral system (Clontech, Mountain view, CA) as described [51]. Transduced cells were selected with hygromycin (200 *μ*g/ml).

### 2.3. Magnetic Sorting and Pre- and Poststaining for CD133 Positivity

Early passages (<20) of CD133(+)/CD133(-) (mixed) parental cells (either BAK, BUL, or STU) were stably transduced with DsRed or GFP (as described above)* prior* to MACS separation according to manufacturer's protocols and antibody (anti-CD133 #130-092- 395, Miltenyi Biotec); CD133(+) cells were further purified over a second MACS® column. After MACS, we had 6 types of cells derived from each line: CD133(+) DsRed, CD133(-) DsRed, CD133(+) GFP, CD133(-) GFP, CD133(+) nonfluorescent, and CD133(-) nonfluorescent. For mixing experiments, we combined red CD133(+) cells and green CD133(-) cells within 24 hours after MACS, and drug treatment was started within 24 hours after that. Within that short time period, CD133 positivity remained relatively constant (Figure 6(e)).

CD133 positivity was always measured after MACS columns; MACS-eluted cell suspensions of either nontransduced, GFP-, or DsRed-expressing “parental” melanoma cells were incubated with either anti-CD133/2 (nontransduced and GFP with Ab clone REA816; Miltenyi Biotec, Auburn, CA) or anti-CD133 (Miltenyi Biotec), followed by Alexa 488 conjugated to goat anti-mouse IgG (for DsRed-expressing cells). Total and viable cell counts were performed by trypan blue staining. CD133(+)/CD133(-) ratios were determined by manual or ImageJ counting of fluorescent Ab-stained cells. Caco2 (ATCC® HTB-37™), a colon cancer line expressing >90% CD133(+), were used as a positive control, while 1205Lu CD133(-) cells served as negative control. Flow cytometry was also performed to confirm the sorted populations using mAb CD133/2-PE (Miltenyi Biotec).

### 2.4. Formation of Melanospheres

Cells were cultured in DMEM/F-12 (1:1) with EGF and FGF (Invitrogen) in plates coated with 10 mg/ml poly(2-hydroxyethyl methacrylate; poly-HEMA) to prevent attachment.

### 2.5. Drug Treatment and Cell Viability Assays

Cells were seeded at 5,000 cells/well in 96-well plates, allowed to recover for 12 h, and exposed to increasing T or D concentrations, alone or in combination, for 72 h. All concentrations of drugs were dissolved in the same volume of DMSO (0.2% [final DMSO]); negative controls also contained 0.2% DMSO. XTT assays were performed to assess cell viability (Biotium, Inc.). Each plate contained the drug-treated cells in triplicate, along with 6 replicates each of 0, 625, 1250, 2500, 5,000, and 10,000 cells in IMDM medium/0.2% DMSO to generate a standard curve of A_450_/min* vs*. cell number (Victor Wallac V3 or EnSpire multimode plate readers (Perkin Elmer)). The standard curve was used to generate the drug dose-response curves. Duplicate plates were used for photomicrographic documentation of cell killing to validate XTT data. Treated parental cells were subjected to XTT cell viability assays and duplicate wells were immunostained with anti-CD133 after 72 h to determine CD133 positivity of the resistant population. Data presented in each figure show mean ± SD of each set of triplicate drug-treated cells from a representative experiment.

### 2.6. Fluorescence Activated Cell Sorting (FACS)

Cells were dissociated from plates by Accutase® and collected by centrifugation. Pellets were incubated with anti-CD133/2-PE (Miltenyi) for 30 min at 4°C, washed with PBST, and diluted in medium to 1x10^7^ cells/ml and then sorted into CD133(+) and CD133(-) subpopulations in 96-well plates containing 5x10^3^ cells per well with a BD FACSAria cell sorter (BD Biosciences; Georgetown Lombardi Comprehensive Cancer Center Flow Cytometry & Cell Sorting Shared Resource).

### 2.7. CD133 Knockdown by siRNA

Knockdown experiments were performed according to standard protocols using small interfering RNAs (siRNAs) specific for CD133 or scrambled siRNA controls (Life Technologies). The sequences used were as shown in Table 1.

### 2.8. Quantitative Reverse-Transcription PCR (qRT-PCR)

Total RNA purified from cell pellets with TRIzol Reagent (Gibco BRL, Grand Island, NY) were subjected to qRT–PCR using two-step reverse transcription–PCR (Invitrogen), 0.75 *μ*g of RNA, and primers (see Table 2).

### 2.9. Statistical Analysis

Assays were performed in triplicate. Error bars are standard deviations of these triplicates and p-values were calculated using a Student's t-test. p values of <0.05 were considered statistically significant. The results are representative of 3 independent experiments with reproducible results. For determining synergism, the combination index (*τ*) was calculated from single dose-response curves and combination experiments as* τ* = *x*_A_/X_A_ + *x*_B_/X_B_, in which, for a given cytotoxic effect, *x*_A_ and *x*_B_ are the concentrations of drugs *A* and *B* in the combination, and X_A_ and X_B_ are the concentrations of drugs* A* and* B* that achieve the same cytotoxic effect when given alone [52]. A *τ* value of 1 indicates additivity, *τ* less than 1 indicates synergy, and *τ* greater than 1 indicates antagonism.

### 2.10. Immunofluorescence

Culture media were removed, and cells fixed with 4% paraformaldehyde, washed in PBST, and incubated 1 hour in Superblock (37515, Thermo Fisher Scientific), followed by primary antibody/Superblock (4°C overnight) against MART-1 (sc-53536, Santa Cruz Biotechnology, 1:100), S100*β* (ab52642, Abcam,1:100). Cells were then incubated with secondary antibodies: Alexa Fluor 488-conjugated goat anti-mouse IgG (ab150113, Abcam, 1:500) or Alexa Fluor 594 goat conjugated anti-rabbit IgG (A11072, Thermo Fisher Scientific, 1:500) for 2 h at room temperature, and then counterstained with DAPI (D1306, Invitrogen, 1:2000) for 20 min. Slides were mounted with ProLong Diamond Antifade (P36961, Invitrogen) for further analysis. Digital images were captured on a Leica SP8 Confocal Microscope. Secondary antibody was utilized as a negative control.

### 2.11. Immunoblot Analysis

SDS–PAGE and transfer of separated proteins to nitrocellulose membranes were performed according to standard procedures. Membranes were stained with Ponceau S (0.1%), to verify equal loading and transfer of proteins, and then incubated with antibodies specific for CD133 (130-092-395, Miltenyi Biotec, 1:1000), MEK1/2 (sc-81504, Santa Cruz Biotechnology, 1:1000), p-MEK1/2 S217/221 (sc81503, Santa Cruz Biotechnology, 1:1000 ), ABCG2 (ab108312, Abcam, 1:1000), and *β*-actin (66009, ProteinTech). Immune complexes were detected by incubation with appropriate horseradish peroxidase-conjugated antibodies to mouse or rabbit IgG (1:3000) and enhanced chemiluminescence (Pierce, Rockford, IL).

## 3. Results

**Figure 1:**
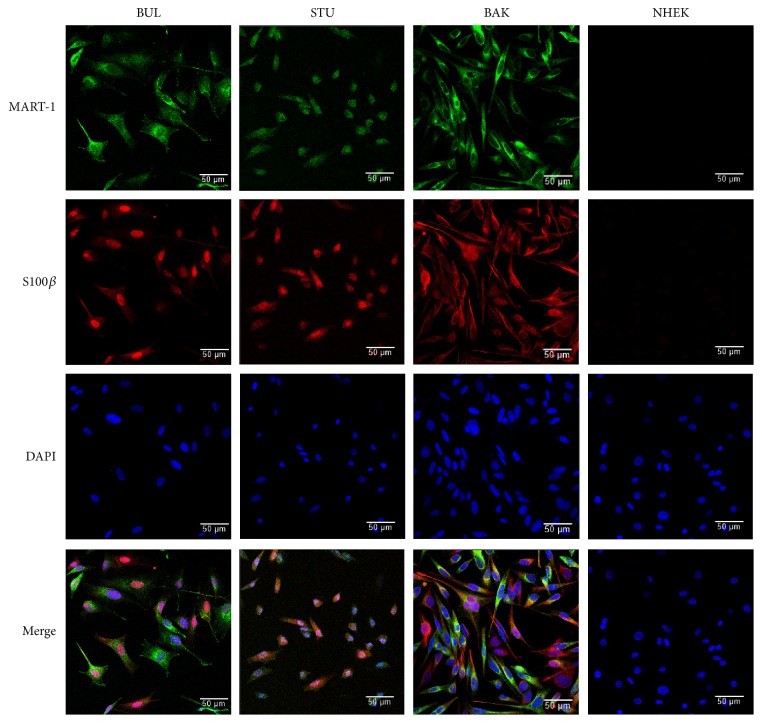
*Patient-derived melanoma cell lines are positive for melanoma markers and exhibit different mutation signatures.* Melanoma identity of parental BAK, BUL, and STU cells was confirmed by immunofluorescence analysis with antibodies against melanocyte markers MART-1 and S-100*β*. Normal human epidermal keratinocytes (NHEK) are included as a negative control. DAPI stain is used to identify nuclei. (Original magnification 20x). The driver mutations are shown in Supplementary Figure s1.

We examined the role of CD133 in chemoresistance. Melanomas were obtained* via *surgical biopsy from fresh lymph node metastases of patients with poor clinical outcomes. Three melanoma cell lines (BAK, BUL, and STU) were established and immunostained for melanocyte-specific antigens MART1 and S100*β* (Figure 1). Specificity was verified by staining a negative cell line (keratinocytes; NHEK), as well as by using secondary antibody alone (Supplementary Figure s1D). BRAF^V600^ or NRAS^Q61^ mutations were determined by Sanger sequencing of PCR products, revealing that BAK and BUL harbor the NRAS^Q61K^ mutation, while STU has the BRAF^V600K^ mutation signature (Supplementary Figures s1 A–C).

### 3.1. Melanoma Cells That Survive T and D Have a Greater Percentage of CD133(+) Cells Than Controls

**Figure 2:**
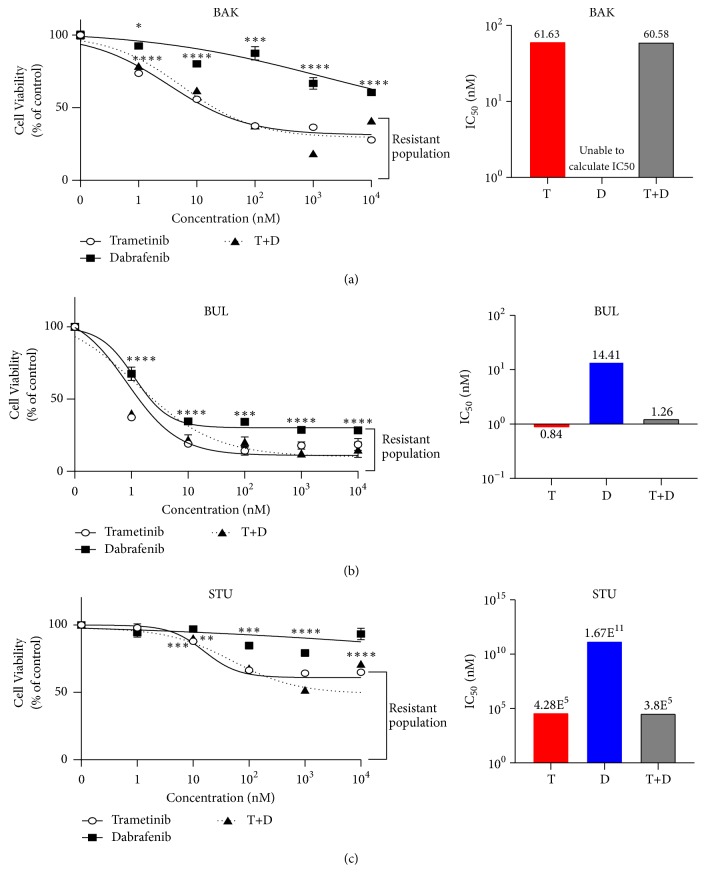
*Patient-derived melanoma cell lines exhibit partial resistance to targeted kinase inhibitors.* Dose-response curves of parental BAK (a), BUL (b), and STU (c) melanoma cells exposed to increasing concentrations (1 nM to 10 *μ*M) of trametinib (T), dabrafenib (D), or both (T+ D). Cells were subjected to XTT cell viability assays 72 h after drug exposure; growth inhibition curves showing percent cell viability relative to control cells exposed to vehicle alone were plotted (left panels), and IC50 was determined (right panels) based on growth inhibition curves. For all experiments *∗*, *∗∗*, *∗∗∗*, or *∗∗∗∗* represent p<.05, <.01, <.001, or <.0001 compared to controls (exposed to vehicle alone); results are the means ± S.D. of three replicates of a representative experiment; essentially the same results were obtained in three independent experiments.

Each patient-derived line was exposed to increasing concentrations of D, T, or a combination of the two drugs. BAK (Figure 2(a)) and STU (Figure 2(c)) cells were partially resistant to dabrafenib. For all lines, T was more effective than D, and the combination of the two was similar to (Figure 2(b)), or more effective than T alone, extending our previous findings with BAK cells [21]. The sensitivities are reflected in the IC50s (Figure 2 right panels). Dose response experiments suggested that certain subpopulation(s) survived high doses of drugs; for example, for all three cell lines, 20-50% of cells survive 10 *μ*M T plus D (Figure 2).

**Figure 3:**
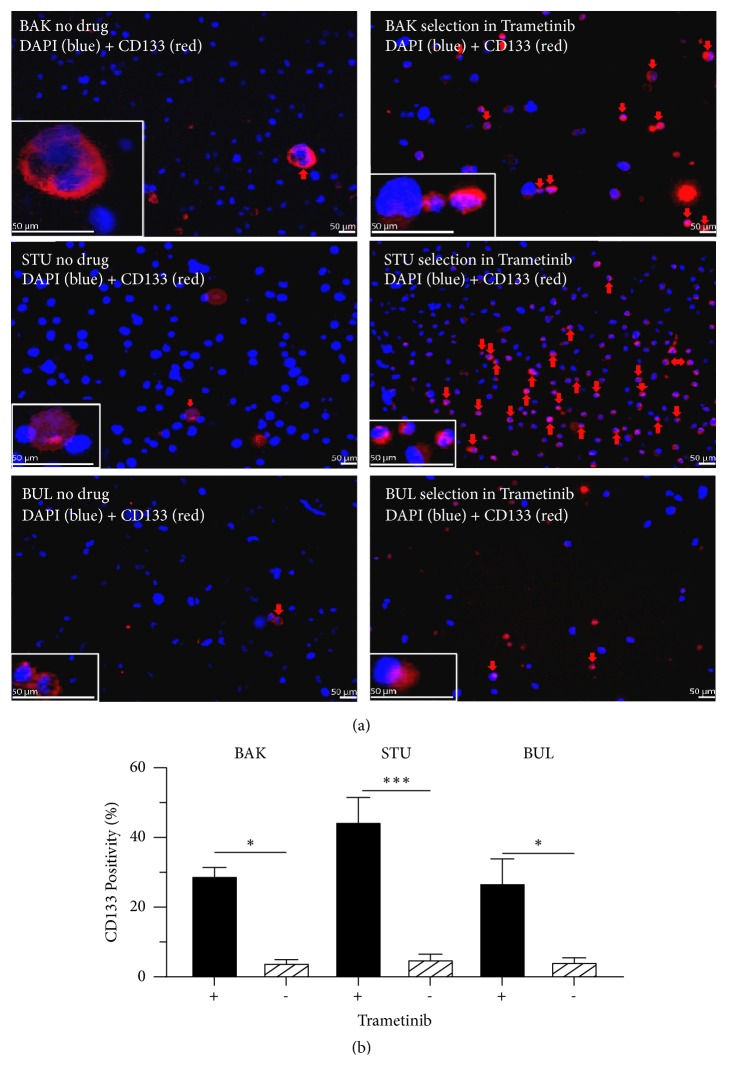
*Surviving populations of BAK, STU, and BUL melanoma cells show increased CD133 positivity.* After cells were exposed to 1 *μ*M of T for 72 h, drug resistant cells were subjected to immunofluorescence staining with PE-conjugated CD133/epitope 2 antibody, counterstained with DAPI, and visualized by fluorescence microscopy. Representative images are shown along with 5-fold magnified insets (lower left of each panel) showing membrane localization of CD133 (a). The percentage of CD133(+) cells (red arrows) in each drug-resistant population was quantified and plotted (b). Error bars represent mean ± SD for triplicates. Experiments were performed three times; a representative experiment is shown.

Since CD133 was shown to be associated with drug resistance and tumorigenicity [53], we determined whether the surviving subpopulations expressed higher levels of CD133, a marker of MIC.  Figure 3(a) shows immunofluorescent staining of each line before or 72 h after drug exposure, and Figure 3(b) shows quantification of CD133(+) cells. ~5% of untreated cells are CD133(+) and significantly enriched after drug treatment (red arrows). In all cases, T significantly increased the proportion of viable cells expressing CD133 (p<0.05).

### 3.2. CD133(+) Cells Enriched by MACS Sorting Exhibit Markers of Melanoma Initiating Stem Cells

**Figure 4:**
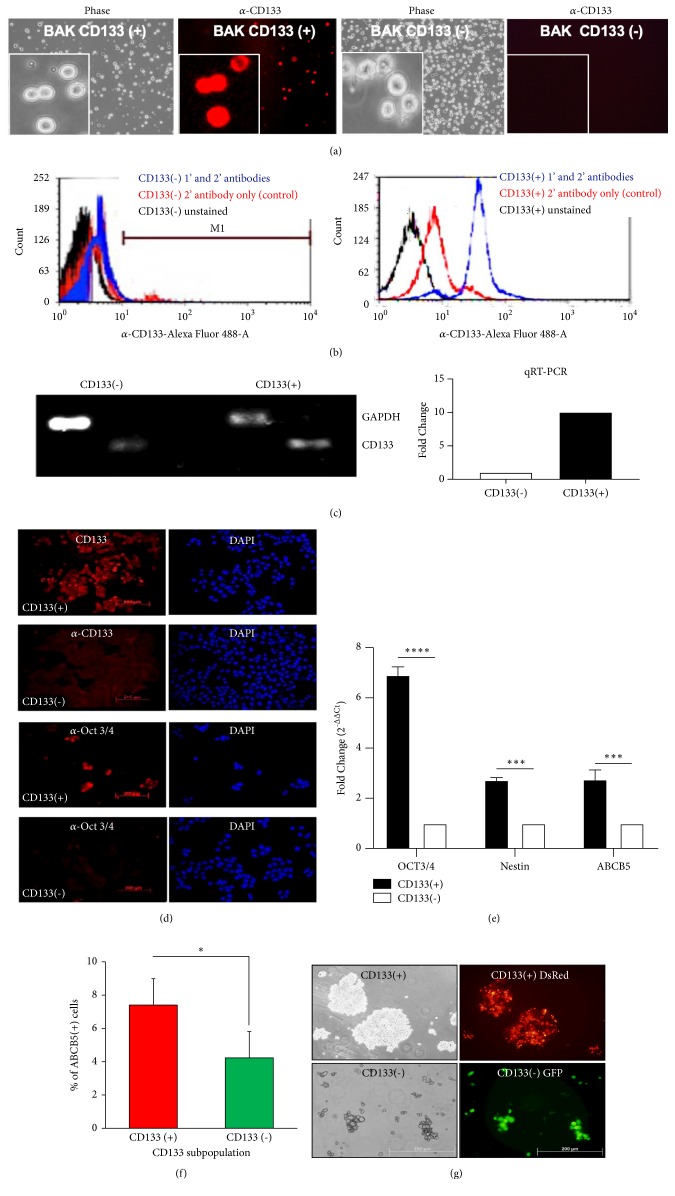
*Magnetically sorted CD133(+) cells exhibit markers of melanoma initiating cells.* BAK cells were separated by MACS and stained for CD133 positivity (a) using CaCo-2 and 1205LU cells as positive and negative controls, respectively (not shown). CD133 positivity was then quantified after imaging with a fluorescence microscope, as well as by flow cytometry (b). (c) RNA from MACS-sorted CD133(+) and CD133(-) BAK cells was subjected to RT-PCR (left) and qRT-PCR (right) analyses for CD133, using GAPDH as a control. (d) Immunofluorescent staining reveals expression of CD133 and stem cell marker Oct 3/4 in CD133(+), but not CD133(-) cells. (e) Additional cancer stem cell markers Nestin and ABCB5 are coexpressed with CD133 and Oct 3/4, using GAPDH as loading control to determine expression levels. (f) Cells were double stained using antibodies specific for ABCB5 and CD133. Cells positive for ABCB5 were quantified in CD133(+) vs. CD133(-) populations. (g) Melanosphere formation in CD133(+) (top), but not CD133(-) cells (bottom).

To determine if the CD133(+) subpopulations consist of MIC, BAK cells were sorted for CD133 positivity using MACS, and examined by immunofluorescence. CD133(+) cells that were retained on the antibody-MACS column stained strongly with anti-CD133/epitope 2-PE (Figure 4(a)). Conversely, CD133 was not detected in CD133(-) MACS column flow-through cells. Flow cytometry (Figure 4(b)) also shows that >90% of MACS column-retained cells are CD133/epitope 2-positive, compared to ~7% of CD133(-) cells. We then determined whether increased CD133 staining was reflected by its RNA levels. Semiquantitative RT-PCR and qRT-PCR revealed that CD133 RNA levels in fact reflect its protein levels, consistent with transcriptional or posttranscriptional regulation of CD133. After MACS sorting, CD133(+) cells exhibited higher levels of CD133 RNA compared to CD133(-) cells as shown by RT-PCR (Figure 4(c) left). qRT-PCR further verified that CD133(+) cells expressed 10-fold more CD133 RNA than CD133(-) cells (Figure 4(c) right). MACS-sorted CD133(+) cells were then examined for the expression of other known cancer stem cell markers. Immunofluorescent staining showed increased expression of Oct3/4 in CD133(+) cells (Figure 4(d)). qRT-PCR of CD133(+) and CD133(-) cells also revealed significant upregulation of the stem cell markers Nestin and ABCB5 along with Oct3/4 and CD133 (Figure 4(e)). To confirm expression of ABCB5 protein in CD133(+) cells, we performed coimmunostaining for both markers, followed by flow cytometric analysis.  Figure 4(f) shows expression of ABCB5 primarily in CD133(+), but not CD133(-) cells. The ability to form spheroids is a hallmark of cancer-initiating cells. DsRed CD133(+) and GFP CD133(-) subpopulations were separated by MACS. CD133(+) cells, but not CD133(-) cells, formed large melanospheres on poly-HEMA (Figure 4(g)). These results suggest that CD133(+) cells comprise the MIC population.

### 3.3. CD133(+) MIC Are More Resistant to T and D

**Figure 5:**
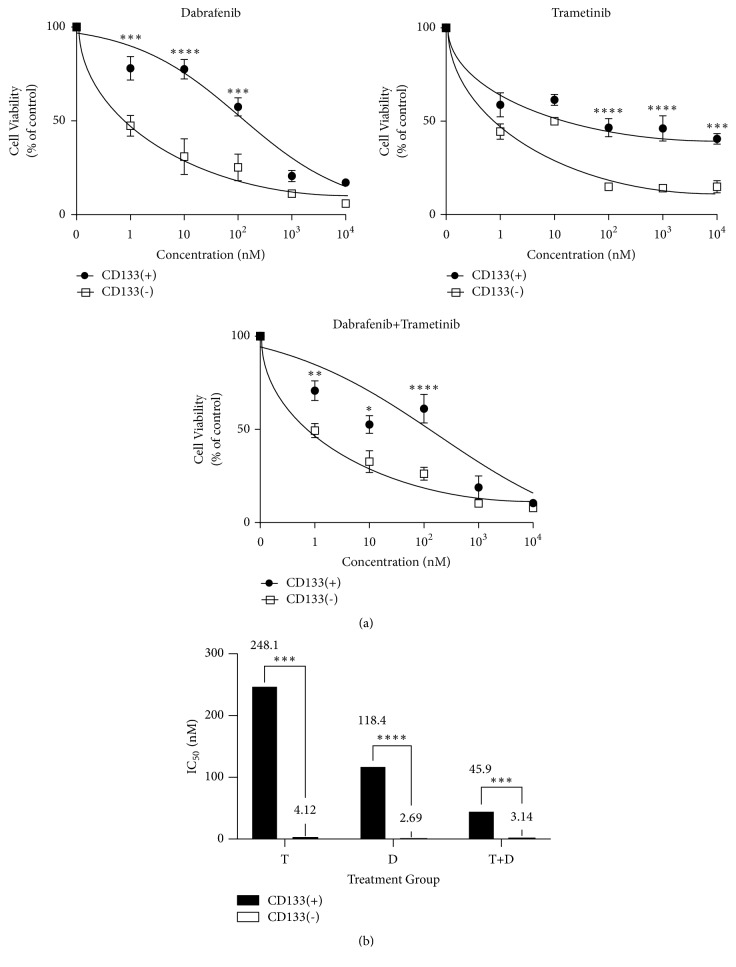
*MACS-sorted CD133(+) BAK melanoma cells are resistant to MAPK inhibitors.* BAK cells were separated by MACS as shown in Figure 4. CD133(+) and CD133(-) BAK cells were then exposed to increasing concentrations MAPKI and cell viability assessed by XTT assays (a). IC50 was then calculated for each treatment group (b). Error bars represent mean ± SD for triplicates. Experiments were performed three times; a representative experiment is shown. Similar results were obtained following FACS sorting of BAK cells (Supplementary Figure s2) or MACS sorting of STU and BUL cells (Supplementary Figure s3).

Increases in CD133(+) cells following drug treatment could result from a phenotypic switch, resistance to drug treatment, or both. To further determine the relative sensitivities of CD133(+) vs. CD133(-) cells to targeted therapeutic kinase inhibitors, MACS-sorted cells were treated for 72 h with D, T, or a combination of the two, and toxicity assessed by XTT. In all cases, CD133(+) cells were significantly more resistant than CD133(-) cells, confirming that CD133 expression is* a priori *predictive of drug resistance (Figure 5).

### 3.4. FACS Sorting Also Reveals Increased Drug Resistance of CD133(+) Cells

BAK cells were separated into CD133(+) and CD133(-) subpopulations by FACS, using anti-CD133-PE, and confirmed by immunofluorescence. 5,000 sorted cells were plated into 96-well plates and then treated for 72 h with D, T, or a combination, to assess drug toxicity. Similar to results obtained by MACS, FACS-sorted CD133(+) cells are more chemoresistant to these mono- or combination therapy treatments, as seen in survival curves (Supplementary Figure s2). FACS thus gives very similar results to those obtained by MACS, confirming that CD133 expression coincides with chemoresistance. Further, combination therapy is the most cytotoxic to CD133(+) cells. CD133 may therefore play a crucial role in drug resistance in melanoma cancer stem cells.

Results were validated with STU (Supplementary Figures s3 A-C), and BUL cells (Supplementary Figures s3 D and E). STU and BUL cells were sorted by MACS, and CD133 positivity confirmed to be more than 85% and less than 10% for CD133(+) and CD133(-), respectively, either by immunostaining (Supplementary Figures s3A and s3D) or flow cytometry (Supplementary Figure s3B). Consistent with BAK, CD133(+) populations from STU (Supplementary Figure s3C) and BUL (Supplementary Figure s3E) cells exhibited significantly greater resistance to D, T, or T+D.

### 3.5. CD133(+) Cells Are Enriched in Mixed Populations by Preferential Survival Rather Than Induction of CD133 in CD133(-) Cells

**Figure 6:**
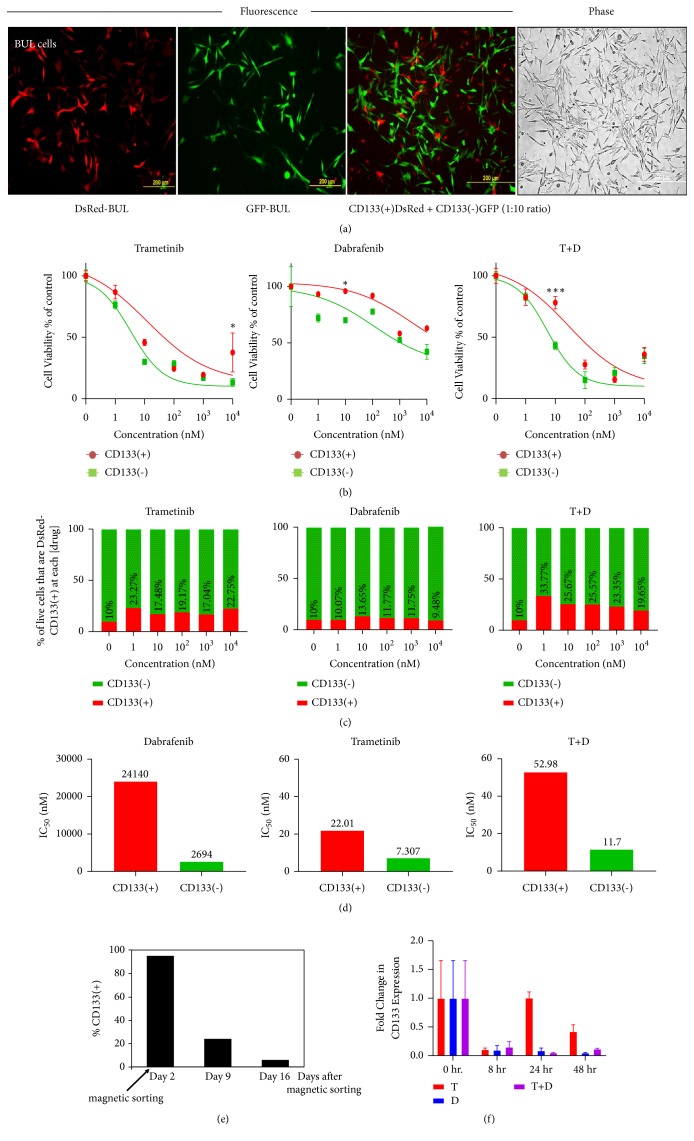
*Mixing experiments with BUL cells show selection for CD133(+) melanoma.* (a) From left: DsRed-expressing BUL CD133(+) cells, GFP-expressing CD133(-) BUL cells, and a 1:10 reconstituted mixture of the two visualized by GFP/DsRed merged fluorescence, and phase contrast microscopy. (b) Dose response of 1:10 reconstituted mixture DsRed-CD133(+) and GFP-CD133(-) subpopulations. The subpopulations were reconstituted in a 1:10 ratio, and mixed cells in triplicate wells were treated with different inhibitor concentrations; fates of each population were monitored by flow cytometry and ImageJ analysis of micrographs. (c) The surviving cells from the two subpopulations are expressed as a fraction of Red CD133(+)/Green CD133(-) at each drug dose. (d) IC50 for each treatment group. (e) MACS-sorted CD133(+) cells were tested for positivity over a 16-day period. (f) BAK cells were exposed to T, D, or T+D, and the levels of CD133 RNA determined by qRT-PCR.

To determine if subpopulations could interconvert, or influence each other's drug susceptibilities, we tagged each cell type and then recapitulated the mixture of cells to trace the fate of each subpopulation throughout the course of the experiments. We derived CD133(+) and CD133(-) BUL melanoma stem/initiating cell subpopulations that express DsRed and GFP, respectively, by stable transduction of BUL cells with retroviral vectors expressing DsRed (LHCX-DsRed) or LHCX-GFP (Figure 6(a), two left panels), followed by MACS sorting. The CD133 positivity of MACS-sorted DsRed cells was confirmed to be approximately 85% by indirect immunofluorescence, whereas the GFP cells exhibited <10% CD133 positivity.

The DsRed-expressing CD133(+) and GFP-expressing CD133(-) subpopulations were mixed in ratios of 1:10 to approximate those present in the parental population (Figure 6(a), two right panels) and then treated for 72 h with T or D, either alone or in combination, to determine drug toxicity using the XTT assay. CD133(-) GFP cells were more sensitive to T, D, and their combination compared to CD133(+) DsRed cells (Figure 6(b)). Accordingly, relative percentages of DsRed-expressing CD133(+) cells showed a dose-dependent increase (Figure 6(c)). The corresponding IC50s are shown in Figure 6(d). It should be noted that the entire period of the experiment was 96 h following MACS sorting, during which time a significantly larger number of CD133(+) cells continued to express CD133, as determined by a time-course experiment in which MACS-sorted CD133(+) cells were tested for positivity over a 16-day period (Figure 6(e)). We obtained the same results using STU (Supplementary Figures s4 A-C) and BAK cells (Supplementary Figure s4D). Thus, while effect of kinase inhibitors for different melanoma cell lines may depend on mutation signatures, CD133 consistently plays a role in chemoresistance in all cell lines examined, even in mixed populations. To further confirm whether CD133 was induced by the drugs administered, BAK cells were exposed to T, D, or T+D, and the levels of CD133 RNA were determined by qRT-PCR.  Figure 6(f) shows that neither T, D, nor T + D increased CD133 RNA levels, further supporting the idea that the fraction of CD133(+) cells increase due to drug resistance, rather than from induction of CD133 (Figure 6(f)).

### 3.6. Potential Mechanisms: Knockdown of CD133 and Inhibition of ABC Transporter

**Figure 7:**
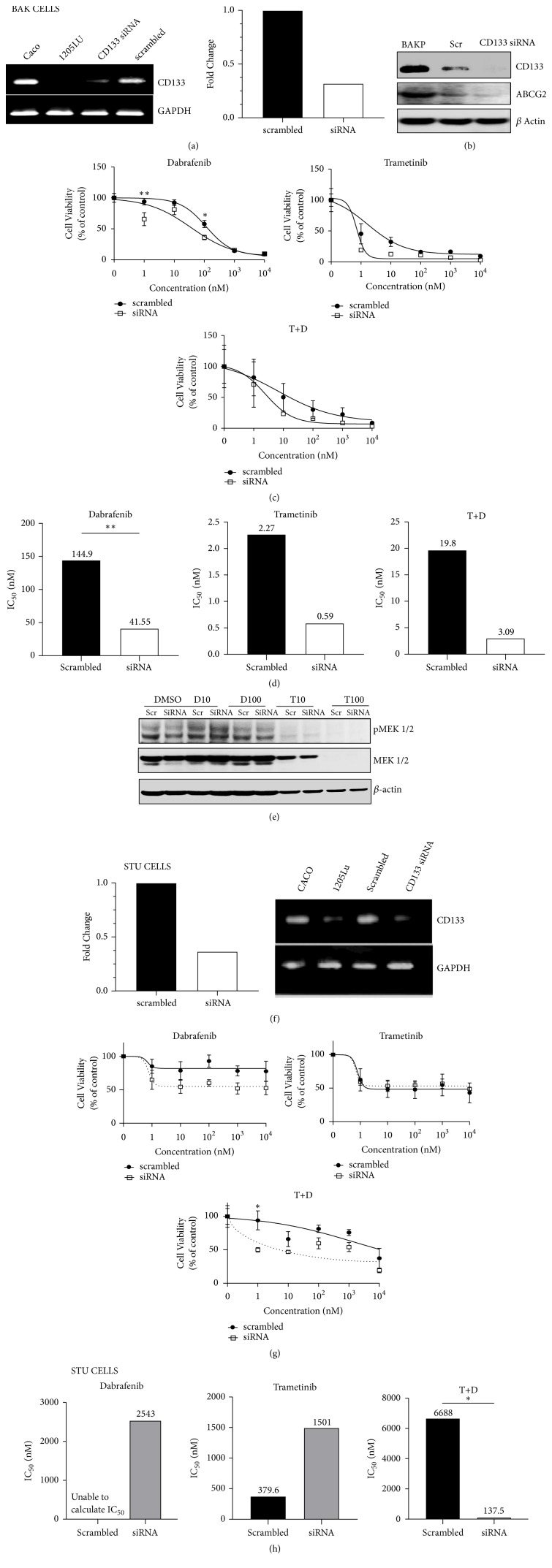
*CD133 siRNA knockdown sensitizes BAK and STU melanoma cells to targeted therapies.* qRT-PCR and RT-PCR of RNA derived from BAK (a) and STU (f) cells after siRNA knockdown of CD133 expression. (b) Immunoblot analysis of CD133, ABCG2, and *β*-actin following siRNA-mediated CD133 knockdown in BAK cells. (c, g) Cell viability (%) of CD133-depleted siRNA knockdown BAK (c) and STU (g) cells compared to scrambled control after exposure to D, T, and the combination treatment. (d, h) IC50s were calculated for BAK or STU cells, respectively. (e) Immunoblot analysis of MEK, and phospho-MEK, following siRNA-mediated CD133 knockdown and exposure to T or D at the indicated doses.

To determine if CD133 is the cause of drug resistance, siRNA knockdown experiments were performed in both BAK and STU cells. CD133 siRNA, but not scrambled control, effectively reduced CD133 RNA levels of BAK and STU cells (Figures 7(a) and 7(f)) by ~70%, diminished BAK CD133 protein levels (Figure 7(b)), and significantly increased the sensitivity of the CD133(+) populations of BAK (Figure 7(c)) and STU (Figure 7(g)), suggesting that CD133 contributes to resistance. Corresponding IC50s for drug-sensitive BAK and STU cells exposed to CD133 siRNA are shown in Figures 7(d) and 7(h), respectively.

**Figure 8:**
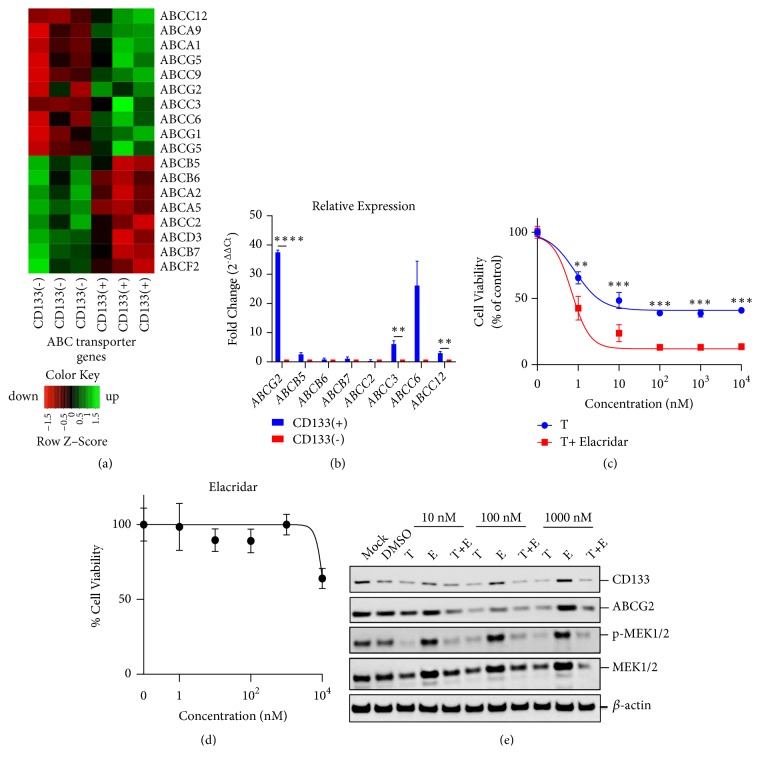
*ABC gene expression contributes to drug resistance of CD133(+) cells.* (a) cDNA Microarray was performed on BAK MACS-sorted CD133(+) and CD133(-) cells using Affymetrix Chip, and raw data was accumulated and converted to normalized data by Partek Genomics Suites. (b) qRT-PCR verified fold change of ABC genes. BAK cells were exposed to the same concentrations of T in the presence or absence of 2 *μ*M elacridar (E; nontoxic concentration; Materials and Methods) and analyzed by XTT (c) or immunoblot analysis specific for phospho-MEK 1/2, total MEK1/2, and actin (e). (d) E dose response of BAK cells.

Microarray analysis of CD133(+) cells revealed a significant (P<0.001) difference in expression of 265 genes compared to CD133(-) cells (manuscript in preparation). A majority are involved in cell cycle regulation and apoptosis; meanwhile, 10 of 18 ABC transporter genes were significantly (P<0.05) upregulated in CD133(+) population, including ABCG2, as determined by microarray (Figure 8(a)) and verified by qRT-PCR analysis (Figure 8(b)), the latter of which revealed a 38-fold upregulation of ABCG2 in CD133(+) BAK cells. While a number of the ABC genes found to be upregulated by microarray were also verified by qRT-PCR, a notable exception was ABCB5, which was determined to be either unchanged or upregulated by microarray. However, qRT-PCR determined that ABCB5 was upregulated 2.75-fold in CD133(+) BAK cells; immunostaining also revealed upregulation of ABCB5 protein in CD133(+) cells (Figure 4(f)).

**Figure 9:**
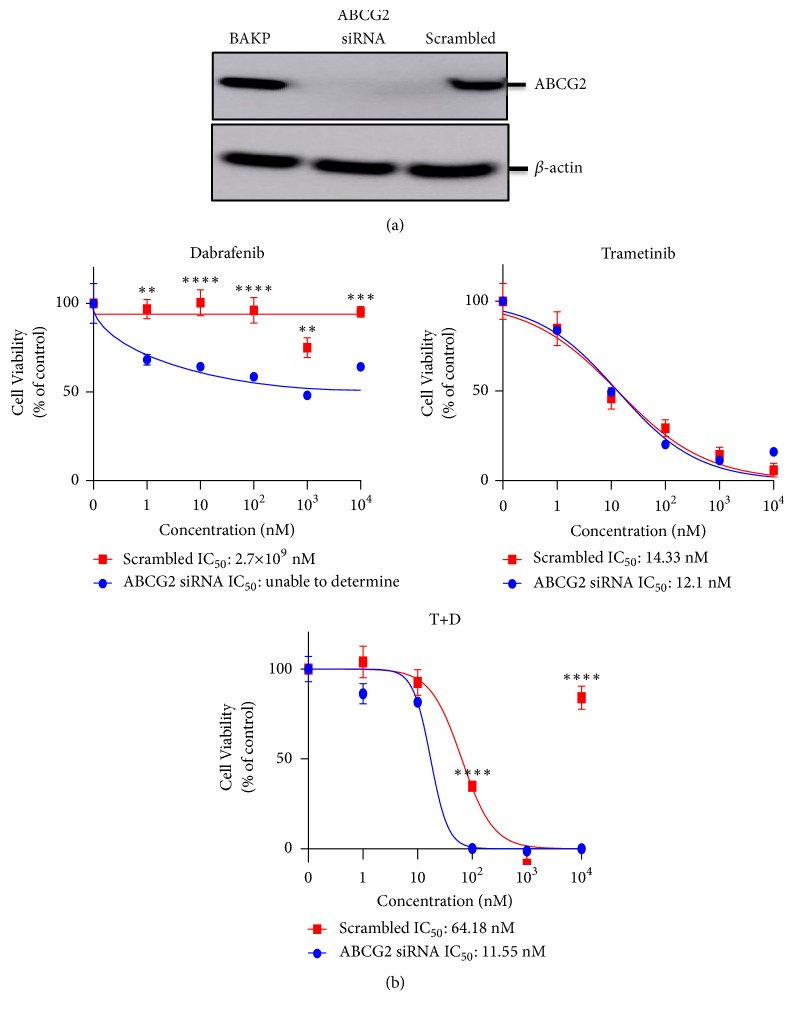
*ABCG2 siRNA knockdown in BAK melanoma cells is sensitized to targeted therapies and increases drug sensitivity of BAK cells.* (a) Immunoblot analysis of ABCG2 and beta-actin following siRNA-mediated CD133 knockdown. (b) Cell viability (%) of CD133-depleted siRNA knockdown BAK cells compared to scrambled control after exposure to D, T, and the combination treatment.

To determine if ABCG2 lies downstream of CD133, BAK cells were exposed to CD133 siRNA and protein levels of CD133, ABCG2, and *β*-actin were determined by immunoblot analysis. Both CD133 and ABCG2, but not *β* actin, were diminished by CD133 siRNA (Figure 7(b)). This suggests that CD133(+) cells may be resistant because they induce expression of ABC gene transporters, such as ABCG2, that play crucial roles in multidrug resistance. Since elacridar (E) is an inhibitor of ABCG2 (as well as ABCB1), we determined the nontoxic concentration that could be used on BAK cells, to examine the role of ABC genes in mediating resistance of CD133(+) cells (Figure 8(d)). Melanoma cells were thus treated with different concentrations of T, in the absence or presence of 2 *μ*M E, a nontoxic concentration. In the presence of E, the efficacy of T was increased synergistically (Figure 8(c); combination index scores [52] <1 for IC30, IC50, and IC70, as shown in Supplementary Table 1) indicating the role of a CD133-ABCG2 pathway in mediating drug resistance, and adding another treatment option to melanoma therapy. Consistently, ABCG2 siRNA knockdown also increased drug sensitivity (Figure 9). No major change in T-mediated MEK1/2 phosphorylation suppression was observed either by T+CD133 siRNA (Figure 7(e), top panel) or by T + E (Figure 8(e)), suggesting either transient unobserved MEK phosphorylation or involvement of additional pathways.

## 4. Discussion

We have shown that CD133 is causally associated with increased resistance of three patient-derived melanoma lines using T, D, or the combination of the two. We verified this several different ways. The first was to expose cells to increasing concentrations of the kinase inhibitors and determine high levels of CD133 expression in surviving cells. The second way was to MACS sort cells into CD133(+) and CD133(-) populations, which revealed chemoresistance in the positive population. The third approach was to sort by FACS, which revealed similar results to MACS. Finally, we knocked down CD133 in all three cell lines and increased drug sensitivity. Together, these results suggest that CD133 confers drug resistance in melanoma cells. It is possible that manipulation of cells alters sensitivity to drugs. Regardless, whenever any of the 3 melanoma cell lines are subjected to the same conditions, CD133(-) cells are more sensitive than CD133(+) cells, even when separated and remixed in a single culture.

We recently performed microarray analysis to uncover potential mechanisms to test (manuscript in preparation). Of note, many of the ABC genes were upregulated (Figure 8). This is consistent with the findings of other investigators, who observed elevated levels of ABC transporters along with CD133 in MIC [21, 23]. Overexpression of CD133 also induced ABCB1 expression and activity leading to drug resistance in glioma [25]. Many of the multikinase inhibitors are substrates of ABCB1, ABCC1, ABCG2, and/or ABCB5. Using* in silico*, cell, and animal models, D and T have been shown to be substrates of ABCB1 and ABCG2 [54, 55]. Interestingly, we found that ABCG2 was upregulated over 35-fold in CD133(+) vs. CD133(-) cells (Figure 8). E, an inhibitor of ABCG2 and ABCB1, also increased BAK drug sensitivity. siRNA-mediated knockdown of ABCG2 markedly inhibited its protein expression (Figure 9(a)), and also increased sensitivity to D and T+D (Figure 9(b)), providing a CD133-ABCG2 pathway as a mechanism for drug resistance.

While ABC gene expression contributes to resistance of CD133(+) cells, another potential mechanism for drug resistance is altered expression of apoptotic or antiapoptotic proteins. We previously showed that T, alone or in combination with mebendazole, induced sub-G1 DNA fragmentation and caspase-mediated PARP cleavage in BAK and BUL cells [56], suggesting that T induces death in part by an apoptotic mechanism. We have also observed apoptotic markers in another NRAS mutant cell line, POT, by T +/- mebendazole (not shown). Therefore, alterations in levels or modification of apoptotic proteins (*e.g.,* Bcl-2 family members) by CD133-mediated pathways may represent a mechanism of resistance.

Finally, increased DNA repair may be a mechanism contributing to drug resistance, as previous studies have shown that ionizing radiation increased the proportion of CD133(+) glioma cells* in vitro* or* in vivo*, probably through increased DNA repair [57]. This study also showed that the increase was primarily due to selective killing of CD133(-) cells, rather than induction of CD133 in CD133(-) cells, which is similar to our findings using sorted melanoma cells. Along with our previous studies [56], our current results suggest that CD133(+) melanoma cells represent a cellular subpopulation that confers melanoma drug resistance and could be a mechanism for survival of melanoma cells, allowing cells to accumulate additional mutations leading to tumor recurrence after therapy. We are currently performing additional studies using* in vivo* models (zebrafish and athymic mouse xenografts) with stable CD133 knockout and inducible lines to further elucidate which of these mechanisms are important in the progression of melanoma.

## 5. Conclusion

Taken together, CD133(+) melanoma cells comprise a subpopulation that confers resistance to multikinase inhibitors currently used in the clinic and may allow melanoma survival and recurrence. Additional studies with stable melanoma CD133 knockout will allow us to target pathways responsible for progression and recurrence of malignant melanoma.

## Figures and Tables

**Table 1:** 

1		Cat #	Sequence
Flexitube siRNA PROM1	1	SI00083741	CACGTTATAGTCCATGGTCCA
Flexitube siRNA PROM1	2	SI00083748	CAGGTAAGAACCCGGATCAAA
Flexitube siRNA PROM1	3	SI00083755	ACCTTTGAGTTTGGTCCCTAA
Flexitube siRNA PROM1	4	SI03098263	CTGGCTAAGTACTATCGTCGA

**Table 2:** 

*Oct4*	forward-5′- CTG GCT TTT CAC TGC TGG CT-3′;
	reverse-5′- TGC TAA GTA GAG TGA ACA GGG-3′;
*Nestin*	forward-5′- CAT TCA GGG AGA CGC CCA-3′;
	reverse-5′- AAC CAC GAC GCC CTT GC-3′;
*CD133*	forward-5′- CCC GGG GCT GCT GTT TAT A-3′
	reverse-5′- ATC ACC AAC AGG GAG ATT G-3′

*GAPDH*	forward-5′-GAA GGT GAA GGT CGG AGT C
*GAPDH*	reverse-5′-C GAA GAT GGT GAT GGG ATT TC

## Data Availability

Data supporting the results reported in a published article can be found within the article, as well as the supplementary material supplied with the article.

## Abbreviations

ABC: ATP-binding cassette transporter family, comprised of 49 genes in humans
ALDH1: Aldehyde dehydrogenase 1
anti-CD133/2: Antibody directed against epitope 2 of CD133 extracellular loop
BMP4: Bone morphogenetic protein 4
*BRAF*: * v-raf* murine sarcoma viral oncogene homolog B
CD133: PROMININ-1
VE-cadherin: CD144/vascular endothelial cadherin
ALCAM: CD166/activated leukocyte cell adhesion molecule
LNGFR: CD271/low affinity nerve growth factor receptor
D: Dabrafenib
E: Elacridar
MIC: Melanoma-initiating cells
NFI: Nuclear factor I
NHEK: Normal human epidermal keratinocyte
*NRAS*: Neuroblastoma RAS viral oncogene homolog
poly-HEMA: Poly(2-hydroxyethyl methacrylate
RP4: Retinitis pigmentosa
T: Trametinib.

## Supplementary Materials

Supplementary Materials
* Supplementary Figure s1: Patient-derived melanoma cell lines exhibit different mutation signatures.* Sanger sequencing analysis of the cell lines established BAK (A) and BUL (B) as BRAF^WT^/ NRAS^Q61K^ and STU120108 (C) as BRAF^V600K^ / NRAS^WT^. (D) Secondary antibody control immunostain of melanoma cells and keratinocytes.* Supplementary Figure s2: FACS-sorted CD133(+) BAK melanoma cells are resistant to MAPK inhibitors.* Dose response curves of BAK cells separated by FACS into CD133(+) and CD133(-) BAK, followed by exposure to increasing concentrations MAPKI and measurement of cell viability by XTT assays (A). IC50 was determined (B) based on growth inhibition curves. Concentration of each drug required to reduce cell number by 50% of maximum inhibition (IC50). T IC50= 96 nM (CD133(+)) vs. 7.1 nM (CD133(-)), dabrafenib 873 nM (CD133(+)) vs. 130 nM (CD133(-)), T + D = 72 nM (CD133(+)) vs. 22 nM (CD133(-)). Error bars represent mean ± SD for triplicates. Experiments were performed three times; a representative experiment is shown.* Supplementary Figure s3: MACS-sorted CD133(+) STU and BUL melanoma cells are resistant to MAPK inhibitors.* STU cells (A-C) and BUL cells (D, E) were separated by MACS and stained for CD133-positivity (A, D), using CaCo2 and 1205LU cells as positive and negative controls, respectively. CD133-positivity was then quantified by flow cytometry with anti-CD133/epitope 2-PE (B). CD133(+) and CD133(-) STU (C) or BUL (E) cells were then exposed to increasing concentrations of T and/or D MAPKI and cell viability assessed by XTT assays. Error bars represent mean ± SD for triplicates. Experiments were performed three times; a representative experiment is shown.* Supplementary Figure s4: CD133 mixing experiments using STU or BAK cells show selection for CD133(+) melanoma.* (A) Positivity of CD133 in Caco-2, 1205LU, and DsRed-CD133(+) and GFP-CD133(-) subpopulations of STU melanoma cells. (B) Merged fluorescent images of mixed CD133(+) (DsRed) and CD133(-) (GFP) BUL subpopulations (Ratio 1:10) without drug or in the presence of 10 *μ*M T, D, or both drugs in combination. (C) Fraction of surviving CD133(+) or (-) cells in triplicate wells treated with increasing inhibitor concentrations. The BUL CD133(+) population shows greater resistance to drug treatments, reflected in the relative increase in DsRed-CD133(+) cell subpopulation. (D) Quantification of BAK cells subjected to mix experiments reveals greater resistance of BAK CD133(+) (DsRed) cells (expressed as percent of surviving DsRed cells in total population). p values of <.05, <.01, or < .001 are shown as one, two, or three asterisks *∗*, respectively.* Supplementary Figure s5: *CD133 expression in melanoma* vs. *days to death. The TCGA database was queried using the UCSC Xena Functional Genomics Explorer (https://xenabrowser.net) and shows a significant negative correlation between CD133 expression and days to death (Kaplan-Meier Estimator).* Supplementary Table 1: *Chou-Talalay analysis of synergistic relationship between T and E.
