# Supplementary material for: CD133 Is Associated with Increased Melanoma Cell Survival after Multikinase Inhibition
Source: J Oncol. 2019 Jul 16;2019:6486173. doi: 10.1155/2019/6486173 (PMC6662463; doi:10.1155/2019/6486173)
Supplement: Supplementary Materials — Supplementary Figure s1: Patient-derived melanoma cell lines exhibit different mutation signatures. Sanger sequencing analysis of the cell lines established BAK (A) and BUL (B) as BRAFWT/ NRASQ61K and STU120108 (C) as BRAFV600K / NRASWT. (D) Secondary antibody control immunostain of melanoma cells and keratinocytes. Supplementary Figure s2: FACS-sorted CD133(+) BAK melanoma cells are resistant to MAPK inhibitors. Dose response curves of BAK cells separated by FACS into CD133(+) and CD133(-) BAK, followed by exposure to increasing concentrations MAPKI and measurement of cell viability by XTT assays (A). IC50 was determined (B) based on growth inhibition curves. Concentration of each drug required to reduce cell number by 50% of maximum inhibition (IC50). T IC50= 96 nM (CD133(+)) vs. 7.1 nM (CD133(-)), dabrafenib 873 nM (CD133(+)) vs. 130 nM (CD133(-)), T + D = 72 nM (CD133(+)) vs. 22 nM (CD133(-)). Error bars represent mean ± SD for triplicates. Experiments were performed three times; a representative experiment is shown. Supplementary Figure s3: MACS-sorted CD133(+) STU and BUL melanoma cells are resistant to MAPK inhibitors. STU cells (A-C) and BUL cells (D, E) were separated by MACS and stained for CD133-positivity (A, D), using CaCo2 and 1205LU cells as positive and negative controls, respectively. CD133-positivity was then quantified by flow cytometry with anti-CD133/epitope 2-PE (B). CD133(+) and CD133(-) STU (C) or BUL (E) cells were then exposed to increasing concentrations of T and/or D MAPKI and cell viability assessed by XTT assays. Error bars represent mean ± SD for triplicates. Experiments were performed three times; a representative experiment is shown. Supplementary Figure s4: CD133 mixing experiments using STU or BAK cells show selection for CD133(+) melanoma. (A) Positivity of CD133 in Caco-2, 1205LU, and DsRed-CD133(+) and GFP-CD133(-) subpopulations of STU melanoma cells. (B) Merged fluorescent images of mixed CD133(+) (DsRed) and CD133(-) (GFP) BU [file 6486173.f1.pdf]

A

Sequencing Results - BAK

Blast

```
Query  35    FFIEDLTVKIGDFGLATVKSRWSGSHQFEQLSGSILWM  148
             F  EDLTVKIGDFGLATVKSRWSGSHQFEQLSGSILWM
Sbjct  583   FLHEDLTVKIGDFGLATVKSRWSGSHQFEQLSGSILWM  620
```

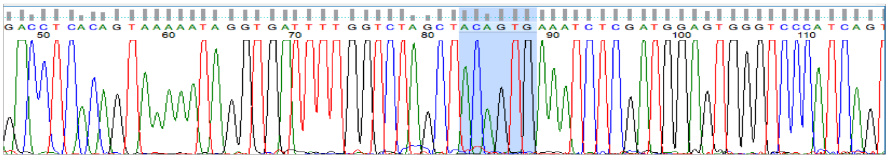

Forward Primer – BRAF

```
Query  157   TSDIFLHEDLTVKIGDFGLATVKSRWSGSHQFEQLSGSILWM  32
             +++IFLHEDLTVKIGDFGLATVKSRWSGSHQFEQLSGSILWM
Sbjct  579   SNNIFLHEDLTVKIGDFGLATVKSRWSGSHQFEQLSGSILWM  620
```

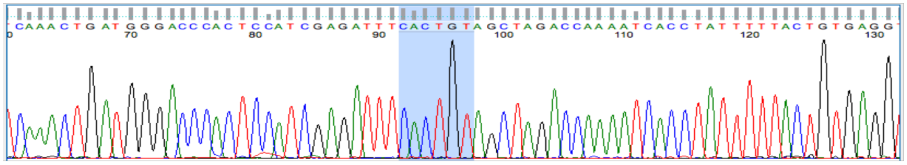

Reverse Primer – BRAF

```
Query  33    CLLDILDTAGQEEYSAMRDQYMRTGEGFLCVFAINNSKSFADIN  164
             CLLDILDTAGQEEYSAMRDQYMRTGEGFLCVFAINNSKSFADIN
Sbjct  51    CLLDILDTAGQEEYSAMRDQYMRTGEGFLCVFAINNSKSFADIN  94
```

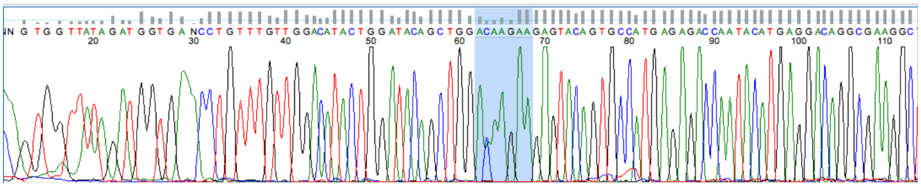

Forward Primer – NRAS

```
Query  164   YPPHPQDSYRKQVVIDGETCLLDILDTAGKEEYSAMRDQYMRTGEG  27
             Y P  +DSYRKQVVIDGETCLLDILDTAG+EEYSAMRDQYMRTGEG
Sbjct  32    YDPTIEDSYRKQVVIDGETCLLDILDTAGQEEYSAMRDQYMRTGEG  77
```

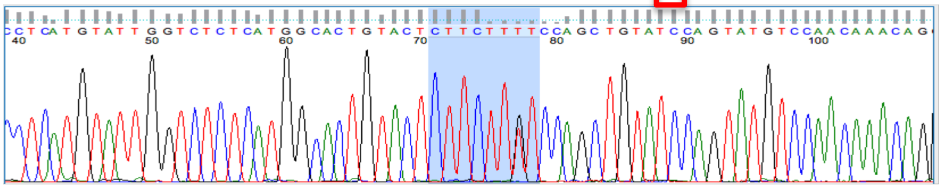

Reverse Primer – NRAS

Cell line	BRAF	NRAS	Condition
BAK	WT	Q61K	Heterozygous - NRAS

Supplementary Figure s1A *BAK cells harbor the NRASQ61K mutation*

B

Blast

Sequencing Results - BUL

```
Query  15   LLXSDIFLHEDLTVKIGDFGLATVKSRWSGSHQFEQLSGSILWM  146
            L  ++IFLHEDLTVKIGDFGLATVKSRWSGSHQFEQLSGSILWM
Sbjct  577  LKSNNIFLHEDLTVKIGDFGLATVKSRWSGSHQFEQLSGSILWM  620
```

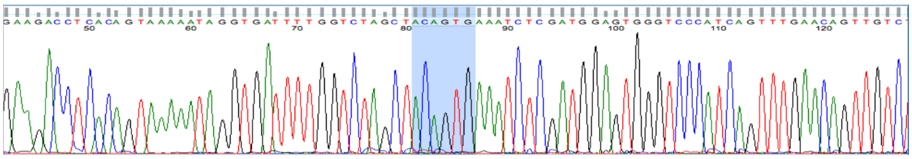

Forward Primer – BRAF

```
Query  159  TSDIFLHEDLTVKIGDFGLATVKSRWSGSHQFEQLSGSILWM  34
            +++IFLHEDLTVKIGDFGLATVKSRWSGSHQFEQLSGSILWM
Sbjct  579  SNNIFLHEDLTVKIGDFGLATVKSRWSGSHQFEQLSGSILWM  620
```

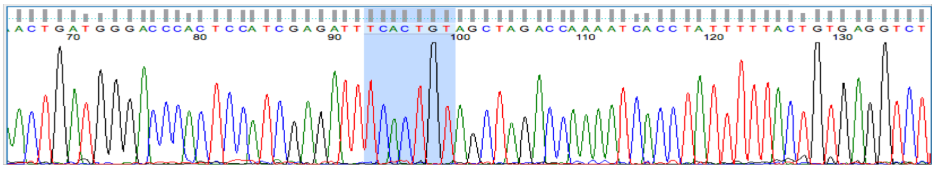

Reverse Primer – BRAF

```
Query  34   CLLDILDTAGQEEYSAMRDQYMRTGEGFLCVFAINNSKSFADIN  165
            CLLDILDTAGQEEYSAMRDQYMRTGEGFLCVFAINNSKSFADIN
Sbjct  51   CLLDILDTAGQEEYSAMRDQYMRTGEGFLCVFAINNSKSFADIN  94
```

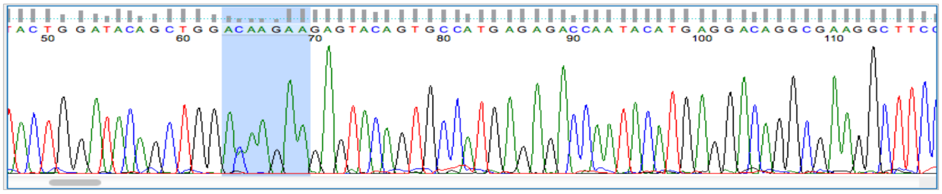

Forward Primer- NRAS

```
Query  165  YPPHPQDSYRKQVVIDGETCLLDILDTAGKEEYSAMRDQYMRTGEG  28
            Y P  +DSYRKQVVIDGETCLLDILDTAG+EEYSAMRDQYMRTGEG
Sbjct  32   YDPTIEDSYRKQVVIDGETCLLDILDTAGQEEYSAMRDQYMRTGEG  77
```

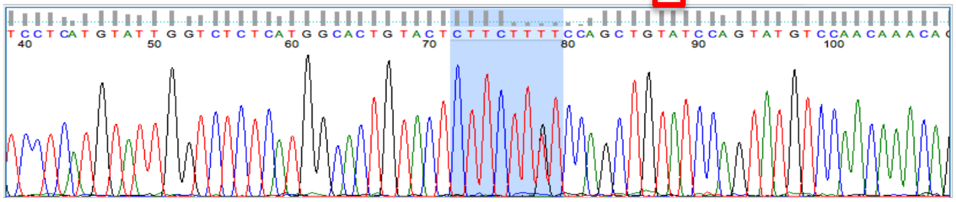

Reverse Primer – NRAS

Cell line	BRAF	NRAS	Condition
BUL	WT	Q61K	Heterozygous - NRAS

Supplementary Figure s1B *BUL cells harbor the NRASQ61K mutation*

C

Blast

Sequencing Results - STU

```
Query   27    SDIFLXEDLTVKIGDFGLATKKSRWSGSHQFEQLSGSILWM   149
              ++IFL EDLTVKIGDFGLAT KSRWSGSHQFEQLSGSILWM
Sbjct   580   NNIFLHEDLTVKIGDFGLATVKSRWSGSHQFEQLSGSILWM   620
```

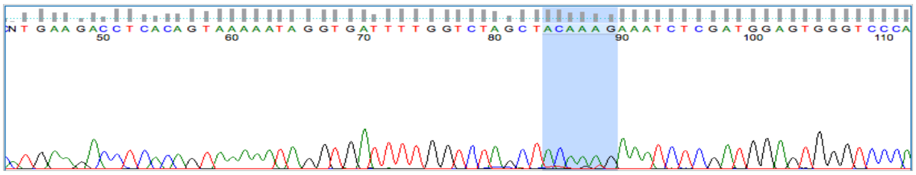

Forward Primer – BRAF

```
Query   156   TSDIFLHEDLTVKIGDFGLATKKSRWSGSHQFEQLSGSILWM----VRMR   19
              +++IFLHEDLTVKIGDFGLAT KSRWSGSHQFEQLSGSILWM    +RM+
Sbjct   579   SNNIFLHEDLTVKIGDFGLATVKSRWSGSHQFEQLSGSILWMAPEVIRMQ   628
```

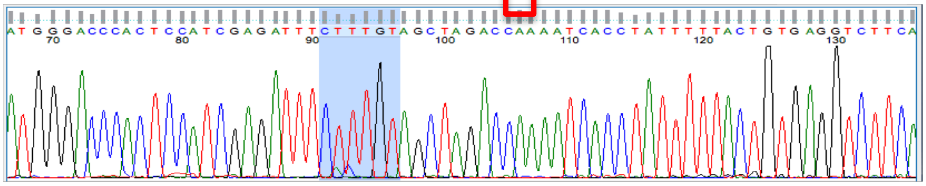

Reverse Primer – BRAF

```
Query   15    VIDGETCLLDILDTAGQEEYSAMRDQYMRTGEGFLCVFAINNSKSFADIN   164
              VIDGETCLLDILDTAGQEEYSAMRDQYMRTGEGFLCVFAINNSKSFADIN
Sbjct   45    VIDGETCLLDILDTAGQEEYSAMRDQYMRTGEGFLCVFAINNSKSFADIN   94
```

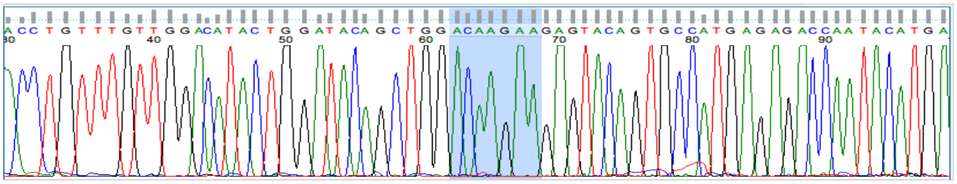

Forward Primer- NRAS

```
Query   162   YPPHPQDSYRKQVVIDGETCLLDILDTAGQEEYSAMRDQYMRTGEG   25
              Y P   +DSYRKQVVIDGETCLLDILDTAGQEEYSAMRDQYMRTGEG
Sbjct   32    YDPTIEDSYRKQVVIDGETCLLDILDTAGQEEYSAMRDQYMRTGEG   77
```

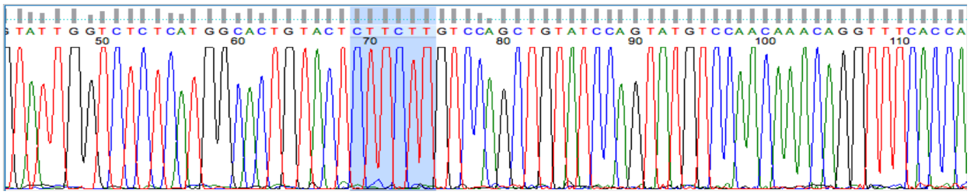

Reverse Primer – NRAS

Cell line	BRAF	NRAS	Condition
STU	V600K	WT	Heterozygous

Supplementary Figure s1C *STU cells harbor the BRAFV600K mutation*

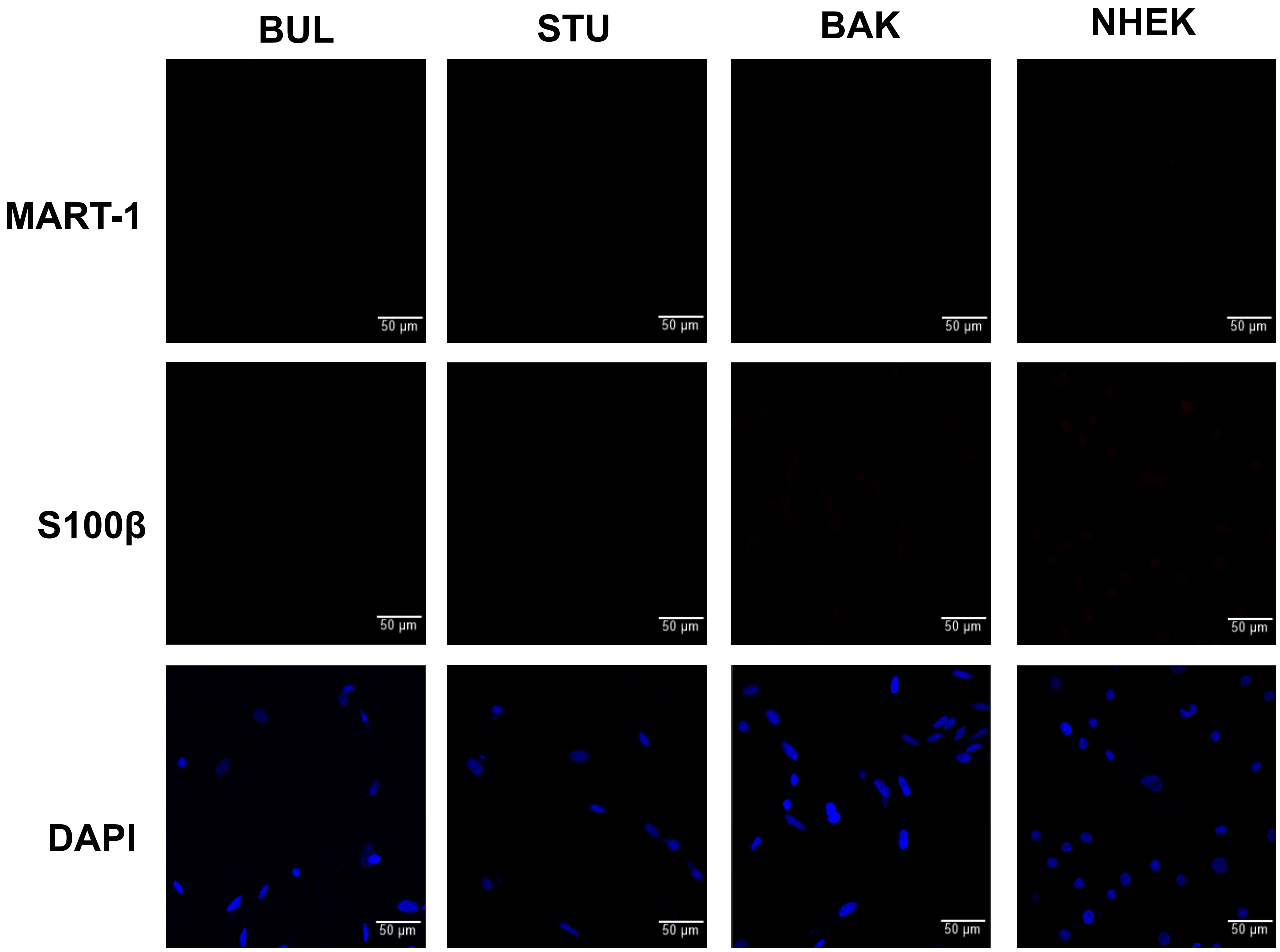

Supplementary Figure s1D *Secondary antibody control for Figure 1*

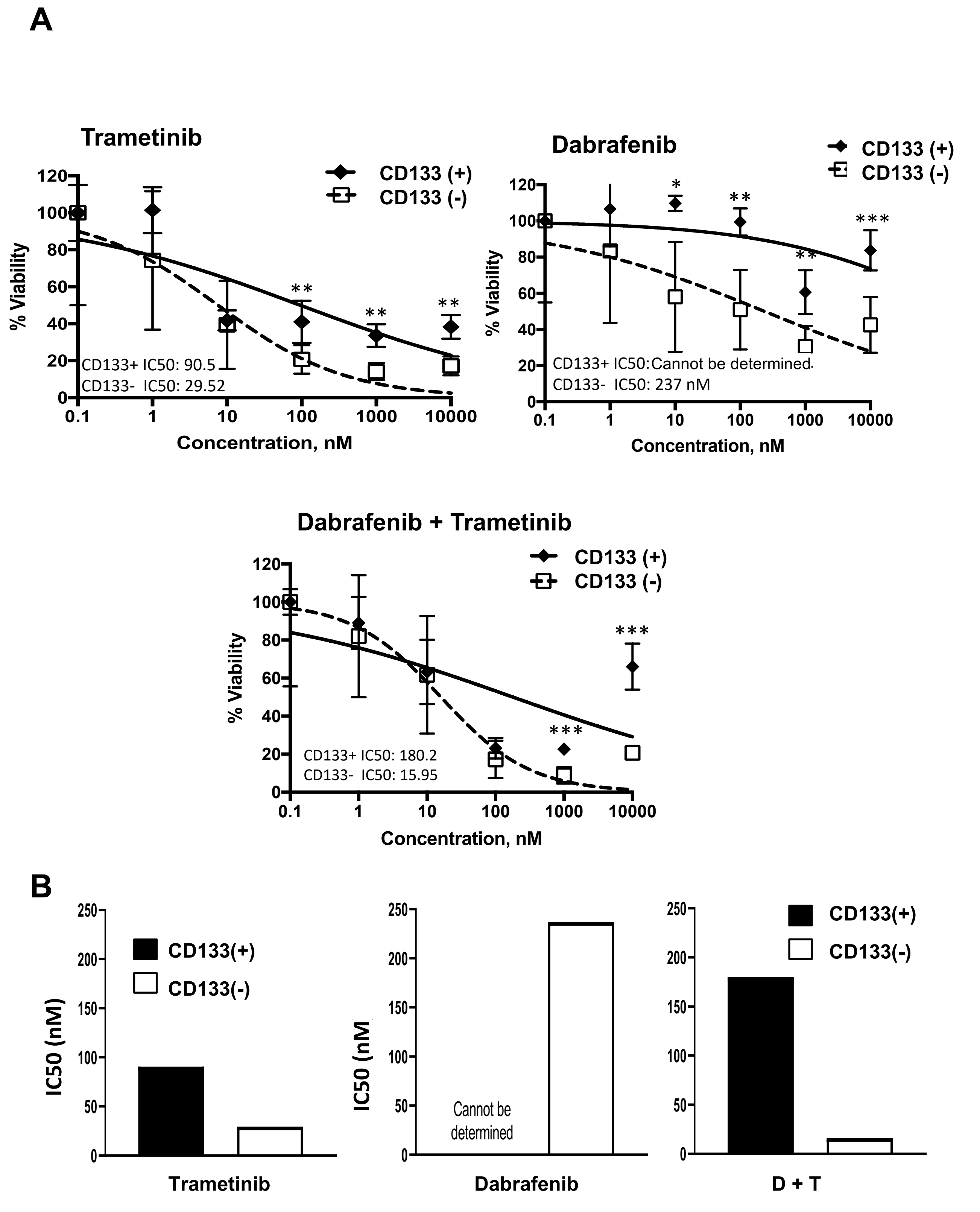

Supplementary Figure s2 *FACS sorting of BAK cells also reveals increased drug resistance of CD133(+) cells. p values of <.05, ,<.01, or < .001 are shown as one, two or three asterisks *, respectively.*

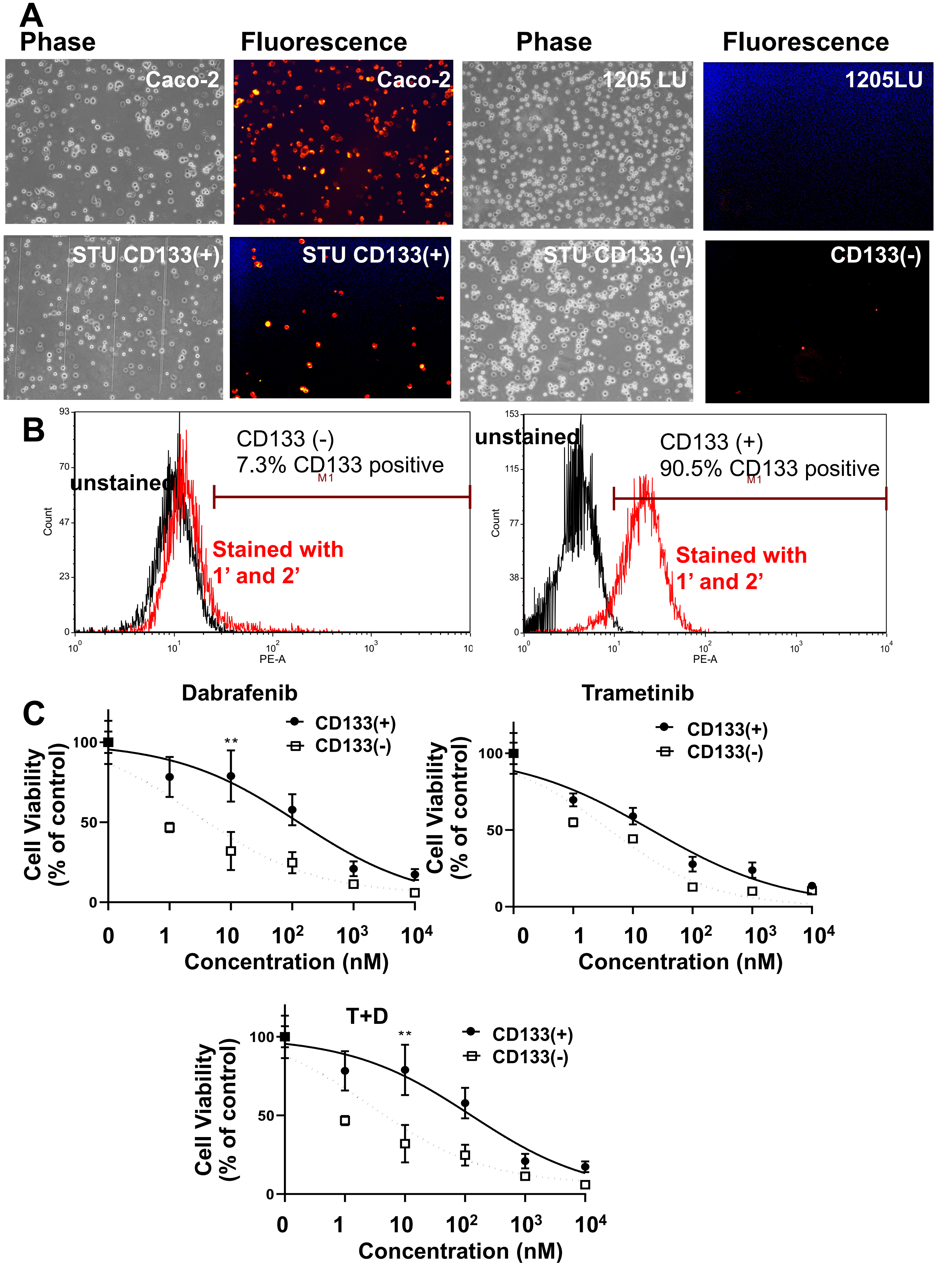

Supplementary Figure s3 A-C. *CD133+ populations from STU cells are drug resistant. p values of <.01 are shown as two asterisks (*), respectively.*

D

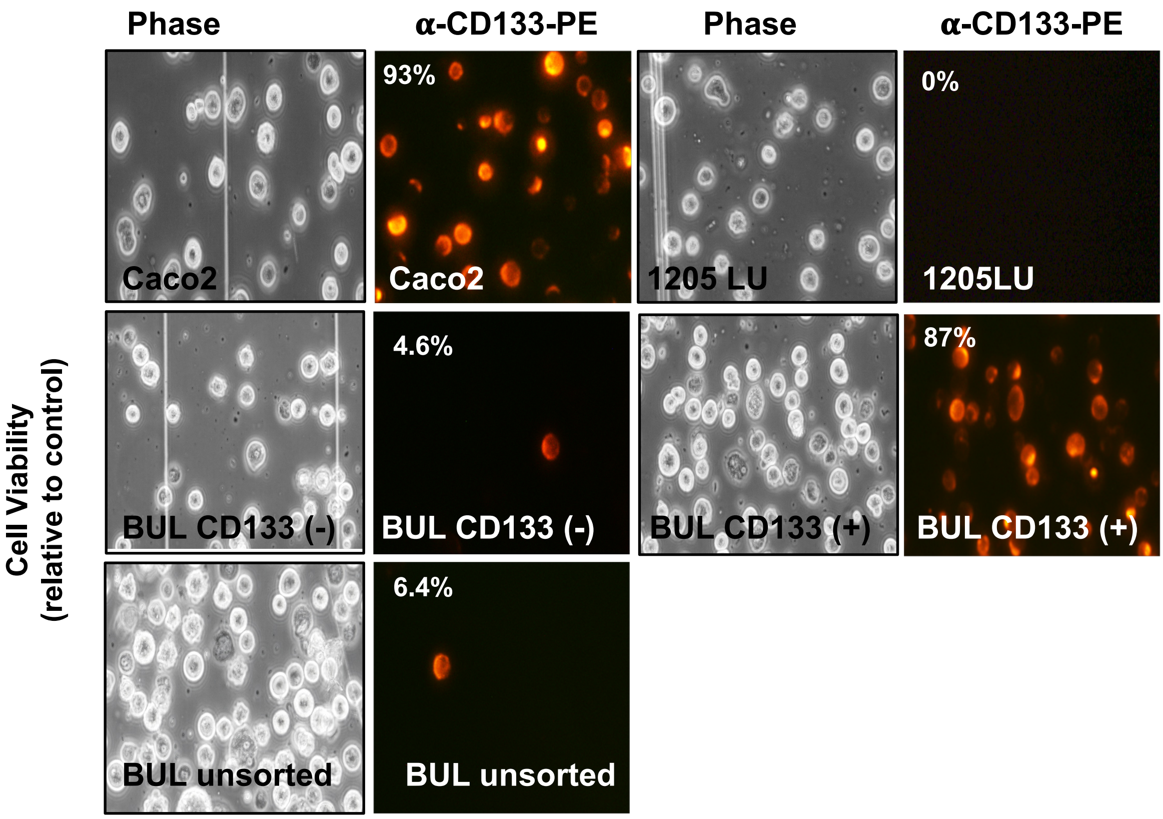

E

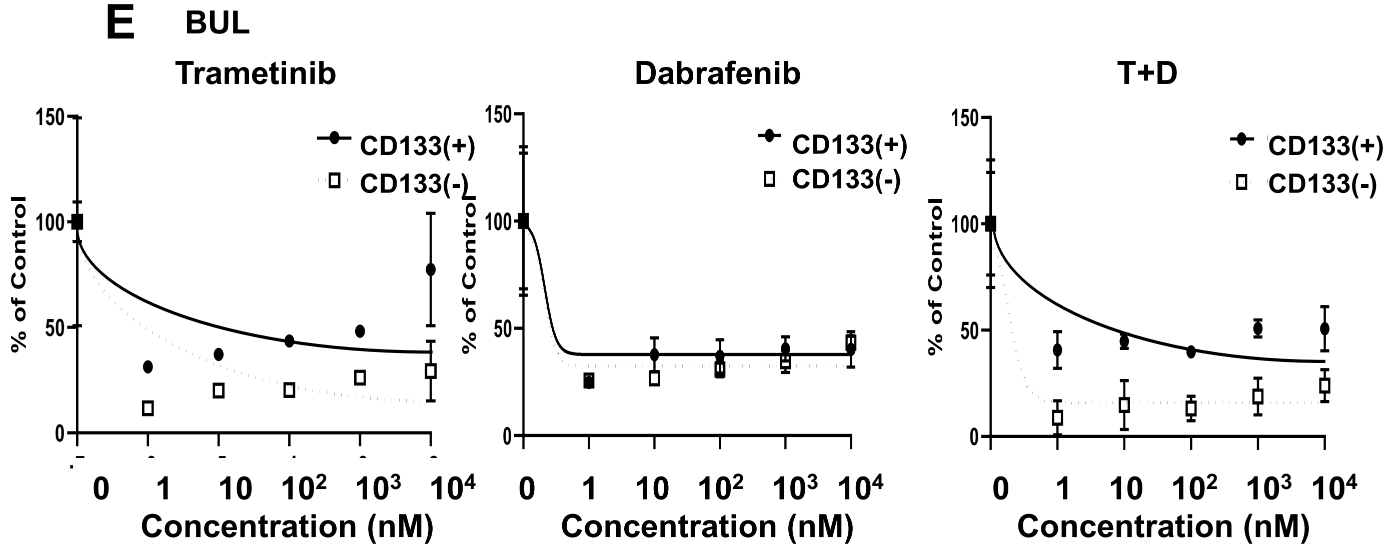

Supplementary Figure s3 D,E. *CD133+ BUL cells are also more resistant to drug treatment. p values of <.05, ,<.01, or < .001 are shown as one, two or three asterisks *, respectively.*

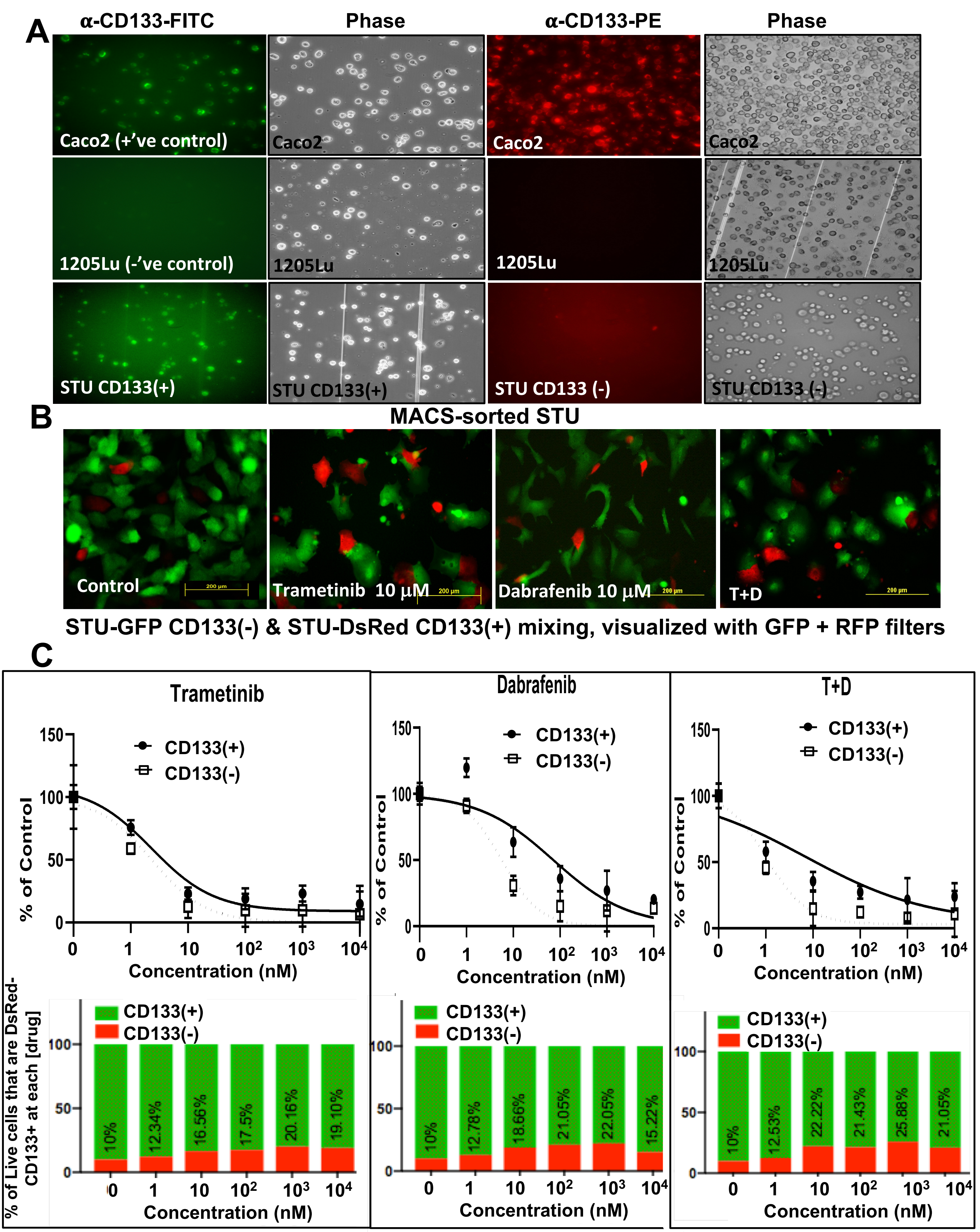

Supplementary Figure s4 A-C. *Stable DsRed CD133+ STU cells preferentially survive drug treatment when reconstituted with GFP CD133- STU cells (thus confirming* ***Figure 6*** *with a second cell line). p values of <.05, ,<.01, or < .001 are shown as one, two or three asterisks *, respectively.*

D

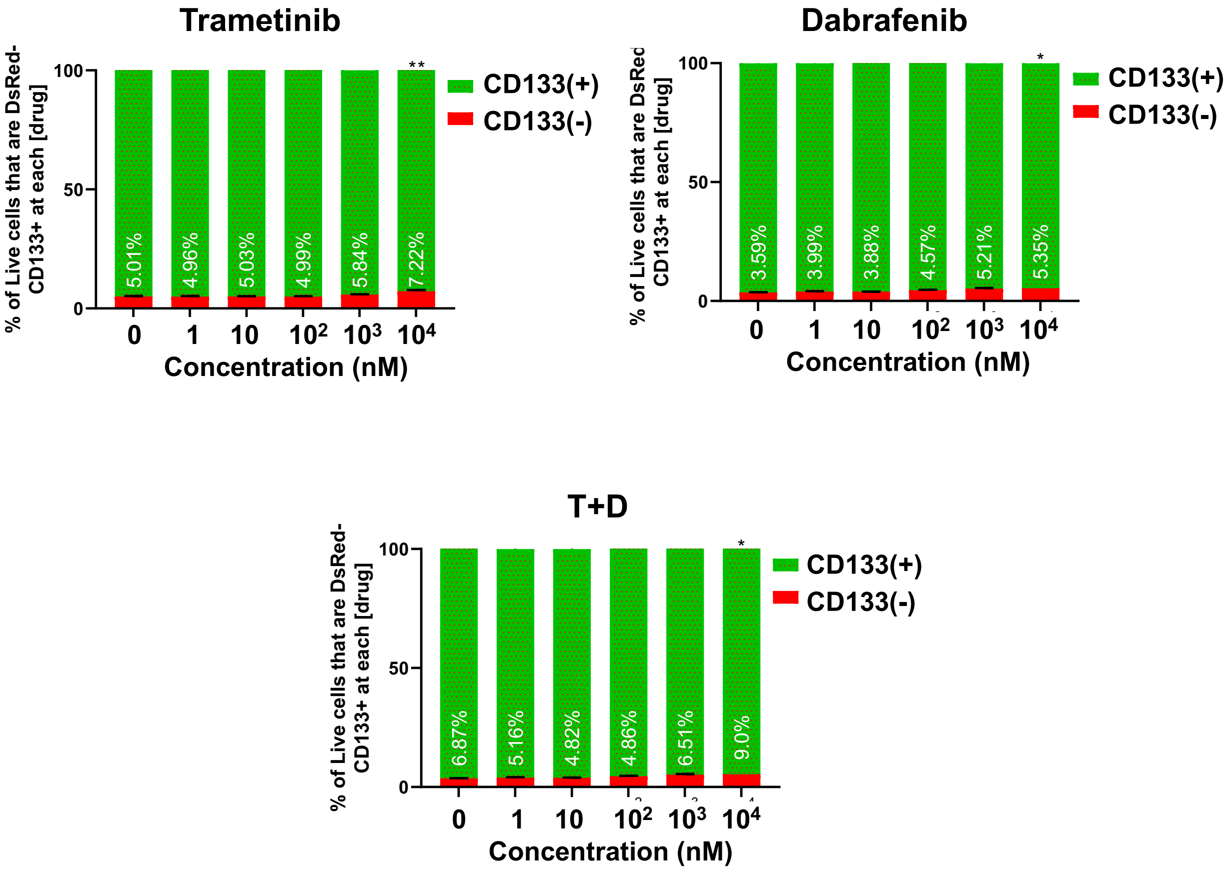

Supplementary Figure s4 D) *Stable DsRed CD133+ BAK cells also preferentially survive drug treatment when reconstituted with GFP CD133- BAK cells (confirming* ***Figure 6*** *with a third cell line). p values of <.05, ,<.01, or < .001 are shown as one, two or three asterisks *, respectively.*

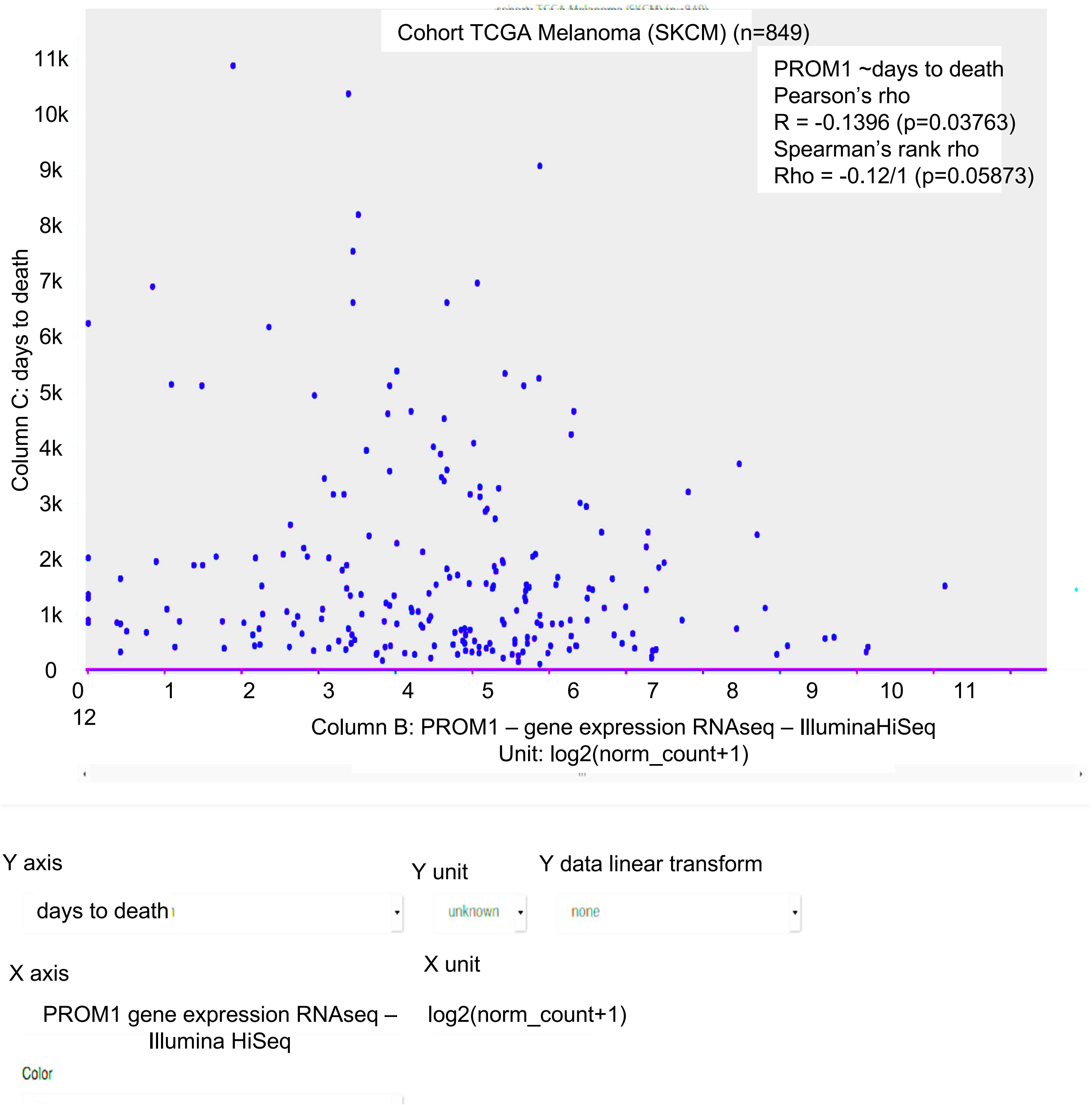

Supplementary Figure s5. *Correlation between CD133 expression in melanoma and days to death (xenobrowser.com)*

Table 1. Combination indices for trametinib plus elacridar

IC	Elacridar (nM)	T (nM)	Combination Index Score	Relationship
30	2000	0.01796	0.238	Synergistic
50	2000	1.326	0.047	Synergistic
70	2000	0.04	0.828	Synergistic

Combination Index of T+E at the IC30, IC50, and IC70 are all <1, therefore the relationship is synergistic.

Supplementary Table 1. *Chou-Talalay analysis of synergistic relationship between Trametinib and Elacridar.*
